# Factors influencing uptake of contraceptive implants in the immediate postpartum period among HIV infected and uninfected women at two Kenyan District Hospitals

**DOI:** 10.1186/s12905-015-0222-1

**Published:** 2015-08-19

**Authors:** Mufida M. Shabiby, Joseph G. Karanja, Francis Odawa, Rose Kosgei, Minnie W. Kibore, James N. Kiarie, John Kinuthia

**Affiliations:** Department of Obstetrics and Gynecology, University of Nairobi, Nairobi, Kenya; Department of Global Health, University of Washington, Seattle, WA USA; Department of Obstetrics and Gynaecology, Kenyatta National Hospital, Nairobi, Kenya

## Abstract

**Background:**

Family planning is a cost effective strategy for prevention of mother to child transmission of HIV and reduction of maternal/infant morbidity and mortality. Contraceptive implants are a safe, effective, long term and reversible family planning method whose use remains low in Kenya. We therefore set out to determine and compare the uptake, and factors influencing uptake of immediate postpartum contraceptive implants among HIV infected and uninfected women at two hospitals in Kenya.

**Methods:**

This cross sectional study targeted postpartum mothers at two Kenyan district hospitals (one urban and one rural). All participants received general family planning and method specific (Implant) counseling followed by immediate insertion of contraceptive implants to those who consented. The data was analyzed by descriptive analysis, T-test, Chi square tests and logistic regression.

**Results:**

One hundred eighty-five participants were enrolled (91 HIV positive and 94 HIV negative) with a mean age of 26 years. HIV positive mothers were significantly older (27.5 years) than their HIV negative counterparts (24.5 years), P = 0.001. The two groups were comparable in education, employment, marital status and religious affiliation. Overall, the uptake of contraceptive implants in the immediate postpartum period was 50.3 % and higher among HIV negative than HIV positive participants (57 % vs. 43 %, P = 0.046). Multivariate analysis revealed that a negative HIV status (P = 0.017) and prior knowledge of contraceptive implants (P = 0.001) were independently associated with increased uptake of contraceptive implants.

**Conclusion:**

There was a high uptake of immediate postpartum contraceptive implants among both HIV infected and un-infected women; efforts therefore need to be made in promoting this method of family planning in Kenya and providing this method to women in the immediate postpartum period so as to utilize this critical opportunity to increase uptake and reduce the high unmet need for family planning.

## Background

Sub-Saharan Africa has the highest burden of adult and pediatric HIV infections globally [1]. The Government of Kenya in collaboration with local and international donors and organizations has made concerted efforts in the prevention, care and treatment of HIV/AIDS with some success. However, HIV prevalence in Kenya is still unacceptably high with a prevalence of 5.6 % among adults with women more likely to be infected (6.9 %) than men (4.4 %) [2]. The prevalence is even higher among pregnant women and stands at 9 % [3]. The second prong of the World Health Organization/United Nations (WHO/UN) strategy on prevention of mother to child HIV transmission (PMTCT) is prevention of unintended pregnancy among HIV infected women [4]. Although family planning is a proven cost effective strategy for achieving Millennium development goals (MDGs) and PMTCT goals and for reducing maternal and childhood morbidity and mortality [4], the rate of unintended pregnancy (26 % for mistimed and 17 % for unwanted pregnancy), and unmet needs of family planning (FP) (25 %) in Kenya still remains high [3, 5].

Contraceptive implants are known to be safe and effective even in the immediate postpartum period, but their use in Kenya remains low (only 1.3 % of the general population currently use it) [4] and its use at the immediate postpartum period is almost negligible.

The study aim was to determine and compare uptake and factors influencing uptake of immediate postpartum contraceptive implant among HIV infected and uninfected women. The findings of this study will assist in identifying acceptability of immediate postpartum contraceptive implant and this in turn has the potential to lead to policy change regarding availability and provision of postpartum implants in our health institutions, thus contributing to reducing the high unmet need of FP and potentially achieve the second prong of PMTCT.

## Methods

### Study design and setting

We conducted a cross sectional study targeting HIV infected and uninfected postpartum women admitted to the postnatal wards (PNW) of two Kenyan district hospitals, Naivasha District Hospital (a rural hospital) and Mbagathi District Hospital (an urban hospital) from the months of July to October 2012. Naivasha and Mbagathi district hospitals are public hospitals that provide both general and specialized care including regular FP service. A room within the postnatal units was used to provide FP counseling and insertion of implants to those who consented.

### Study population

Study participants were postpartum mothers in the postnatal wards of the two hospitals after delivery and before discharge. One HIV positive mother was recruited for every one HIV negative mother. The HIV status was obtained from the patients’ ANC card during admission into the postnatal wards and then confirmed from the patients’ hospital files. Those with unknown HIV status, were tested in the maternity unit and results documented in the patients’ files. The enrollment criteria included; consenting adult postpartum women with known HIV status, normotensive and with no known serious medical conditions such as active liver disease, deep venous thrombosis, migraine with aura, renal failure or breast cancer.

### Ethical considerations

Ethical approval was obtained from the Kenyatta National Hospital/University of Nairobi (KNH/UON) Ethics and Research committee. Consent was obtained in writing from all the study participants after adequate explanation for enrollment in this study. The study was conducted using an anonymous survey with no name identifying information provided in the questionnaire. All measures to maintain anonymity and confidentiality were strictly followed.

### Data collection and recruitment

**Fig. 1:**
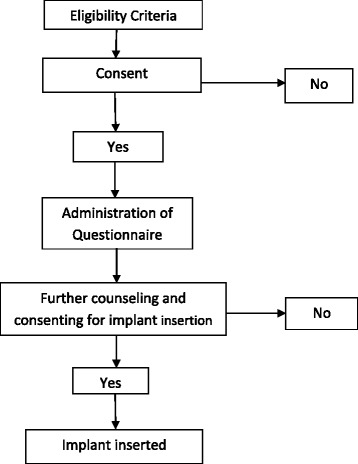
Algorithm for enrolment of study participants

Participant enrollment into the study was carried out by convenience sampling based the admission of patients in the PNW until the numbers of HIV positive and negative balanced. Pretesting of the questionnaire was done at the study sites using the same protocol and no revision was required. Trained research nurses administered the structured questionnaire and the information collected included; socio-demographic characteristics, parity, contraceptive knowledge and use, spouse approval of contraceptive use, future fertility intentions and knowledge and opinions about contraceptive implants. This was then followed by standardized contraceptive counseling about all available FP methods and specifically on the study method (implants). Those who further consented to insertion of contraceptive implants were inserted immediately after signing part B of the consent form and were encouraged to continue using barrier methods. A card that indicated the method given, date inserted and expiry date was given together with post insertion instructions on wound care. They were then advised to go to FP clinic of their choice for follow up. Those who did not consent to implant insertion were counseled on importance of FP use and referred to FP clinics of their choice for routine postpartum care (Fig. 1).

### Data analysis

Descriptive statistics were used to describe baseline maternal characteristics such as age, parity, education levels and FP knowledge. Mean and median was used for continuous variables and frequencies, and proportions were used for categorical variables. T-test was applied to compare means, while a two-sided chi-square test was used to compare proportions between the two groups. Fisher’s exact test was also utilized when at least one cell had a value of zero. Logistic regression was then applied to test the strength of association between demographic and reproductive variables and the primary outcome – uptake of contraceptive implants. The significant factors were then subjected to multivariate analysis to analyse for independent association.

The data was entered into password protected Microsoft Access Database and subsequently transferred to SPSS statistical package for analysis. SPSS 16.0, Inc., 2007.

### Study limitations

One of the study limitations was selection bias, due to the non-probability sampling method but this was mitigated by the large sample size.

## Results

### Socio-demographic characteristics

185 participants were enrolled - 91 HIV positive and 94 HIV negative. The socio-demographic profiles of the HIV positive and negative participants were similar (Table 1). Majority of the participants were in their mid-twenties and married, while close to half had completed primary education and were employed.

**Table 1:** Socio-demographic characteristics of participants by HIV status

Variable	HIV Status		P value
	HIV Positive (n = 91) N (%)	HIV Negative (n = 94) N (%)	
Age			
Average age (years) ± SD	27.5 ± 5.4	24.5 ± 5.2	<0.001
Study site			
Naivasha D.H	47 (51)	46 (49)	0.71
Mbagathi D.H	44 (48)	48 (52)	
Formal education			
Primary	50 (48.5)	53 (51.5)	0.84
Secondary/tertiary	41 (50)	41 (50)	
Marital status			
Married	71 (46.1)	83 (53.9)	0.06
Not married	20 (64.5)	11 (35.5)	
Occupation			
Employed	45 (48)	48 (52)	0.83
Not employed	46 (50)	46 (50)	

### Reproductive characteristics

Majority of the participants had attended ANC (96 %), however HIV positive mothers were significantly more likely to have had a discussion with a health care worker at the ANC than their HIV negative counterparts (P = 0.002), [Table 2]. HIV positive mothers regarded the ideal family size as having fewer children compared to HIV negative mothers, however they were more likely to report higher order pregnancies. There was also a high overall rate of unintended pregnancy of 44 % among all participants and this was significantly higher in HIV positive (53 %) compared to HIV negative (36 %) mothers (P = 0.024), [Table 2].

**Table 2:** Reproductive health characteristics of participants by HIV status

Variable	HIV status		Total N (%)	P value
	HIV+ (n = 91) N (%)	HIV - (n = 94) N (%)		
ANC attendance				
Yes	87 (48.9)	91 (51.1)	178 (96.2)	0.482
No	4 (57.1)	3 (42.9)	7 (3.8)	
FP discussion during ANC				
Yes	64 (58.2)	46 (41.8)	110 (59.5)	0.002
No	27 (36)	48 (64)	75 (40.5)	
Ideal family size, Mean (SD)	2.9 (0.8)	3.3 (0.9)	3.1 (0.9)	0.007
Number of pregnancies				
Gravida 1	16 (29.1)	39 (70.9)	55 (30)	<0.001
Gravida 2	29 (53.7)	25 (46.3)	54 (29)	0.44
Gravida 3 and above	46 (59)	30 (41)	76 (41)	
Choice of pregnancy				
Planned	43 (47)	60 (64)	103 (56)	0.024
Unintended	48 (53)	34 (36)	82 (44)	

### Contraceptive knowledge

Most participants were aware about modern methods of FP with injectable contraceptives(93 %) and contraceptive pills (92 %) being the most commonly known FP methods, followed by IUCD (65 %), male condom (62 %) and implants (61 %) as shown in Table 3. Natural FP methods such as Lactational Amenorrhoea (LAM) (3 %) and rhythm methods (2.7 %) were among the least known FP methods. Only about a quarter of all participants knew about female sterilisation (29 %). Significantly more HIV positive women knew about sterilization methods than HIV negative women [Table 3].

**Table 3:** Knowledge of FP methods among HIV positive and negative women

Knowledge of FP methods	HIV status		Total N (%)	P value
	Positive N (%)	Negative N (%)		
IUCD	65 (71.4)	55 (58.5)	120 (65)	0.066
LAM	2 (2.2)	4 (4.3)	6 (3.2)	0.430
Rhythm method	2 (2.2)	3 (3.2)	5 (2.7)	0.677
Female condom	11 (12)	18 (19.1)	29 (15.7)	0.187
Female sterilization	39 (42.9)	15 (16)	54 (29.2)	<0.001
Implants	62 (68.1)	51 (54.3)	113 (61.1)	0.053
Injectable	84 (92.3)	88 (93.6)	172 (93)	0.728
Male condom	64 (70.3)	50 (53.2)	114 (61.6)	0.017
Male sterilization	11 (12.1)	3 (3.2)	14 (7.6)	0.022
Contraceptive pills	85 (93.4)	85 (90.4)	170 (92)	0.458
Withdrawal method	16 (17.6)	13 (13.8)	29 (15.7)	0.483

### Uptake of contraceptive implant

The overall uptake of immediate postpartum contraceptive implants was 50.3 % [Table 4], however HIV positive mothers were less likely to accept implant insertion than their HIV negative counterparts (OR 0.91 [95 % CI: 0.82-0.998], P =0.046). Table 4 also describes the uptake of implants by HIV status, whereby 43 % and 57 % of HIV positive and negative mothers respectively accepted immediate postpartum implants insertion.

**Table 4:** Uptake of immediate postpartum contraceptive implants among HIV infected and Uninfected women at Mbagathi and Naivasha District Hospitals

Variable	Postpartum contraceptive implants uptake		OR [95 % CI]	P value
	Yes	No		
Overall uptake	93 (50.3 %)	92 (49.7 %)		
HIV status				
Positive	39 (42.9 %)	52 (57.1 %)	0.91 [0.82-0.99]	0.046
Negative	54 (57.4 %)	40 (42.6 %)	Ref	

### Factors influencing uptake

As demonstrated in Table 5, the uptake of immediate postpartum implants was higher in postpartum mothers who were from Mbagathi District Hospital and unemployed; however none of these associations were statistically significant. Interestingly, mothers’ education level or marital status did not influence implants uptake. HIV status influenced uptake of postpartum implants with fewer HIV positive mothers accepting implants insertion compared to HIV negative with a trend towards significance, (OR 0.6 [95 % CI: 0.3-1.0], P = 0.047). Similarly, those with spouse approval were twice as likely to accept postpartum implants than those without spouse approval (OR 2.0 [95 % CI:1.1-2.4], P = 0.02), while those with prior knowledge of contraceptive implants were three times more likely to accept implants insertion compared to those who had no prior knowledge of implants (OR 3.3 [95 % CI:1.5-7.4], P = 0.001). Similarly, those of younger age at first pregnancy (19.5 years) were also more likely to accept postpartum implants compared to those of older age at first pregnancy (20.6 years) (P value = 0.01). On multivariate analysis, independent factors found to be associated with increased uptake of implants were; negative HIV status and prior implant knowledge. Younger age at first pregnancy and having spouse approval were not independently associated with uptake of postpartum implants.

**Table 5:** Factors influencing uptake of implants among all participants

Variable	Implants uptake		OR [95 % CI]	P value
	Yes (n = 93) N (%)	No (n = 92) N (%)		
Average age ± SD	25.7 ± 5.7	26.4 ± 5.2	NA	0.33
Study Site				
Naivasha D.H	43 (46.2)	50 (53.8)	0.7 [0.4-1.3]	0.27
Mbagathi D.H	50 (54.3)	42 (45.7)	1.0	
Formal education				
Primary	53 (51.5)	50 (48.5)	1.1 [0.6-2.1]	0.72
Secondary/tertiary	40 (48.8)	42 (51.2)	1.0	
Marital status				
Married	78 (50.6)	76 (49.4)	1.1 [0.5-2.6]	0.82
Not married	15 (48.4)	16 (51.6)	1.0	
Occupation				
Employed	43 (46.2)	50 (53.8)	0.7 [0.4-1.3]	0.270
Unemployed	50 (54.3)	42 (45.7)	1.0	
HIV status				
Positive	39 (42.9)	52 (57.1)	0.6 [0.3-1.0]	0.047
Negative	54 (57.4)	40 (42.6)	1.0	
Choice of pregnancy				
Planned pregnancy	53(51)	50(49)	1.1 [0.6-1.9]	0.72
Unintended pregnancy	40(49)	42(51)	1.0	
Ideal family size (Mean ± SD)	3.05 ± 0.8	3.12 ± 0.9	NA	0.61
Male birth				
Yes	44 (48.9)	46 (51.1)	0.8 [0.4-1.5]	0.44
No	47 (54.7)	39 (45.3)	1.0	
Gravida				
Gravida 1-2	56 (51.4)	53 (48.6)	1.1 [0.6-2.1]	0.72
Gravida 3 and above	37 (48.7)	39 (51.3)	1.0	
Spouse approval				
Yes	73(54.5)	61(45.5)	2.0 [1.1-2.4]	0.02
No	11(55)	9(45)	1.0	
Jadelle knowledge				
Yes	80 (57.1)	60 (42.9)	3.3 [1.5-7.4]	0.001
No	13 (28.9)	32 (71.1)	1.0	
Age at first pregnancy				
Mean ± SD	19.5 ± 2.7	20.6 ± 3.7	NA	0.013
Plans for resumption to sexual				
Activity in weeks (Mean ± SD)	11.75 ± 9.8	9.7 ± 7.10	NA	0.156

On subgroup analysis by HIV status and factors influencing uptake of implant, none of the socio-demographic factors significantly influenced uptake. However, on multivariate analysis, independent factors found to be associated with increased uptake of implants among HIV positive mothers were spouse approval and resumption of sexual activity. Spouse approval was positively associated with increased uptake of postpartum implants; while plans for early resumption of sexual activity were negatively associated with the uptake. On the other hand, in HIV negative mothers, those with prior knowledge of implants were threefold more likely to accept postpartum implants than those without prior knowledge. Early age at first pregnancy was not significantly associated with increased uptake.

### Reasons for declining uptake

**Fig. 2:**
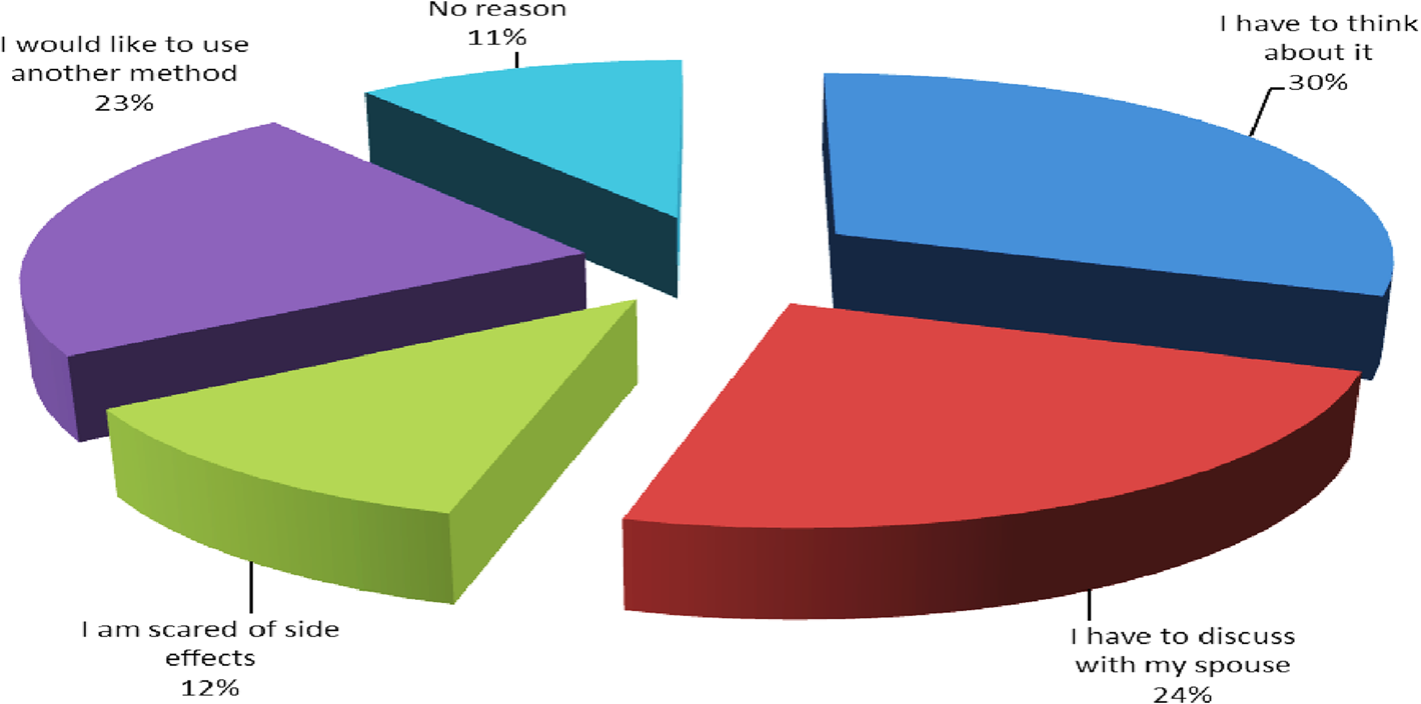
Reasons for declining postpartum implants among all clients

Among all clients, the most common reason for declining postpartum implants was “I have to think about it” (30 %) followed by “I have to discuss with my spouse” (24 %) and “I would like to use another method” (23 %). Some had no reason (11 %) while 12 % were afraid of side effects [Fig. 2].

**Fig. 3:**
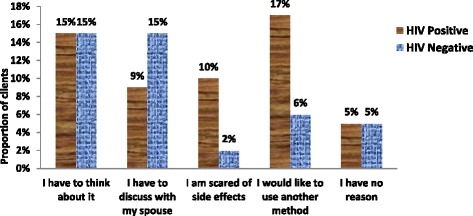
Reasons for declining postpartum implants by HIV status, P value = 0.039

However, there were twice as many HIV negative women compared to HIV positive women who wanted to discuss contraceptive implant uptake with their spouse. On the other hand as shown in Fig. 3, HIV positive mothers were five times more likely than HIV negative to report being afraid of side effects and three times more likely to report that they wished to use another method. The difference in the reasons given for declining postpartum implants between the two groups were statistically significant (P = 0.039). There were similar proportions of HIV positive and negative clients who declined postpartum implants because they had to think about it (15 %) or had no reason of declining (5 %).

## Discussion

In this study we demonstrated a good uptake of postpartum implants at 50.3 %, with the uptake being higher among the HIV negative (57 %) than the HIV positive clients (43 %). Although we demonstrated an uptake higher than that found by Dhont et al. in Rwanda (38 %) [6] - who looked at effect of improved access on the uptake of Long Acting Reversible Contraceptives (LARC)- both studies show that improving access to LARC improves their uptake particularly in regards to implants. The uptake in our study is much higher than the current use of contraceptive implants in Kenya where only 1.3 % of the general population is currently using it [5]. Currently, immediate postpartum contraceptive implants are not routinely offered in the postnatal wards of our health care system and are not part of the national FP guideline [7]. Despite proven safety in the use of immediate postpartum contraceptive implants, [8] the Kenya national guidelines on FP which are based on the WHO medical eligibility criteria (MEC), [9] have restricted its use in breastfeeding mothers to 4 weeks postpartum and allowed for its immediate postpartum use only in non-breastfeeding mothers [7]. In view of the high uptake we found when we offered contraceptive implants immediately postpartum, there is need to change this policy so as to improve access to this contraceptive method among both HIV positive and negative women. This will go a long way in reducing the unmet need of FP, high rate of unintended pregnancies and mother to child transmission of HIV. Hopefully the new WHO MEC expected this year will have these changes.

Prior knowledge of implants positively influenced uptake among all participants with those who knew about implants being three times more likely to accept them. This finding is consistent with that of Tanfer who found that interest in the use of implants increased with its knowledge [10]. Similarly, Jacobstein and Stanley attributed the upswing of use of contraceptive implants in Eastern and South African countries in particular Ethiopia and Rwanda to the marked rise in implants knowledge [11]. Educating the public on this method will result in a ripple effect of improving uptake, reducing unintended pregnancies and PMTCT.

On the other hand, spouse approval significantly influenced uptake among all participants and especially among HIV positive clients. Similar findings were found by Bii et al. [12] and Akelo et al. [13], who found that partner involvement and approval was crucial in FP decision making in HIV positive mothers. Mutiso et al. also found that marital status and having a regular sexual partner influenced contraceptive use [14]. This finding emphasizes the importance of individual and couple FP counseling to improve FP uptake and hence achieve PMTCT.

The findings of this study on the factors that influence uptake of contraceptive implants, concur with the argument of Johnson et al. [15] that HIV positive women exhibit fertility desires and contraceptive behavior that are different from other women, and therefore, more needs to be done to understand their specific needs and concerns in order to reduce the unacceptably high rate of unintended pregnancy and achieve the second prong of PMTCT. As demonstrated in this study, different factors influenced uptake of immediate postpartum implants differently among HIV positive and negative mothers. In addition the HIV positive women were more concerned about the potential side effects of implants; this is consistent with the findings of Credé et al. who also found that HIV positive women were concerned of the safety of hormonal FP methods given their health status [16]. Therefore, more needs to be done to understand these factors that influence FP uptake among both HIV positive and negative mothers so that appropriate strategies are put in place to reduce unmet needs of FP.

The main reason for declining to implants insertion among all clients was; wanted to think about it, need to discuss with spouse and wanted to use another method, but among HIV positive clients there was also the fear of side effects. In view of this, proper education on implants as an FP method and its effect on HIV as well as emphasis on spouse involvement needs to be given to this subgroup in order to allay any fears and improve its acceptance.

Consistent with the findings of Peltzer et al. [17] who looked at FP needs of PMTCT clients in resource poor settings in South Africa, we also found high fertility desire among HIV positive clients where more than a half of HIV infected women (67 %) regarded three or more children as ideal family. Similarly, more than a half of HIV positive clients reported higher order pregnancies of 3 and above compared to their HIV negative counterparts. This is not surprising considering they were significantly older than the HIV negative women in the study. This may also explain the ‘replacement phenomenon’ described by Magadi and Agwanda in which HIV positive women tend to get pregnant more frequently so as to replace any child loss resulting from the anticipated increased infant/child mortality [18].

Prong 2 of PMTCT is elimination of unintended pregnancy. This study found an overall rate of unintended pregnancy of 44 %, and 60 % among the HIV positive clients in comparison to Kenya Demographic and Health Survey (KDHS) 2009 that reported an unintended pregnancy rate of 26 %. This finding is consistent with other studies which have shown that HIV positive women are more likely to report higher rates of unintended pregnancies [17–19]. This can be explained by the fact that HIV positive women regarded 1–2 children as ideal family size yet majority reported their index pregnancy to be of higher order (gravida 3 and above). This study finding has reaffirmed the need for stakeholders to strengthen the 2nd prong of PMTCT.

## Conclusion

The high rates of unintended pregnancies especially among HIV positive women is disturbing and this indicates missed opportunity for PMTCT and hence the need to scale up the second prong of PMTCT.

Postpartum contraceptive implants are an acceptable, effective and safe FP method and should be considered to be offered routinely to both HIV positive and negative postpartum mothers.

Ministry of Health through the Division of Reproductive Health need to develop policies and review FP guidelines to allow use of implants in the postpartum period so as to increase its uptake in tandem with its strategic plan on long acting and reversible contraceptive methods.

There is a need to develop communication and advocacy strategies to increase public awareness of this method to improve uptake. Integration of FP services to HIV care and other reproductive health services will improve access and should also emphasize on partner participation in decision making. Concerns among HIV positive women of potential side effects are valid and health education strategies need to be developed to allay fears and improve understanding of this method of FP.
